# Co-infection of vvMDV with multiple subgroups of avian leukosis viruses in indigenous chicken flocks in China

**DOI:** 10.1186/s12917-019-2041-3

**Published:** 2019-08-13

**Authors:** Tuofan Li, Jing Xie, Guangcheng Liang, Dan Ren, Shu Sun, Lu Lv, Quan Xie, Hongxia Shao, Wei Gao, Aijian Qin, Jianqiang Ye

**Affiliations:** 1 Key Laboratory of Jiangsu Preventive Veterinary Medicine, Key Laboratory for Avian Preventive Medicine, Ministry of Education, College of Veterinary Medicine, Yangzhou University, No. 12 East Wenhui Road, Yangzhou, Jiangsu 225009 People’s Republic of China; 2 Jiangsu Co-innovation Center for Prevention and Control of Important Animal Infectious Diseases and Zoonoses, Yangzhou, 225009 Jiangsu China; 3 Joint International Research Laboratory of Agriculture and Agri-Product Safety, the Ministry of Education of China, Yangzhou University, Yangzhou, 225009 Jiangsu China; 4 Institutes of Agricultural Science and Technology Development, Yangzhou University, Yangzhou, 225009 Jiangsu China

**Keywords:** MDV, Multiple ALV, Co-infection, Molecular analysis

## Abstract

**Background:**

In China, although the ALV eradication program and the MD vaccination strategy greatly reduce the disease burdens caused by the infection of ALV and MDV, the frequent emergence of novel ALV-K or vvMDV in the vaccinated chicken flock challenges the current control strategies for both diseases.

**Results:**

In Guangdong Province, an indigenous chicken flock was infected with neoplastic disease. Hematoxylin–eosin staining of the tissues showed the typical characteristics of MDV and classical ALV infection. The PCR and sequencing data demonstrated that the identified MDV was clustered into a very virulent MDV strain endemic in domestic chickens in China. Moreover, subgroups ALV-A and ALV-K were efficiently recovered from two samples. The full genome sequence revealed that the ALV-K isolate was phylogenetically close to the ALV TW3593 isolate from Taiwan Province.

**Conclusions:**

A co-infection of vvMDV with multiple ALV subgroups emerged in a chicken flock with neoplastic disease in Guangdong Province. The co-infection with different subgroups of ALV with vvMDV in one chicken flock poses the risk for the emergence of novel ALVs and heavily burdens the control strategy for MDV.

## Background

Avian leukosis virus (ALV) and Marek’s disease virus (MDV) are the most causative agents for neoplastic disease in chickens [1]. ALV is currently classified into seven subgroups (A-E, J and K) in chickens based on the antigenicity of its envelope protein [2]. Infection with avian leukosis virus subgroup A (ALV-A) or ALV-B generally results in classical lymphocytic leukemia, while ALV-J infection mainly causes myeloid leukosis and vascular neoplasms [3–5]. ALV-C and ALV-D are rare in clinical cases, whereas ALV-K is a novel subtype of ALV recently identified in indigenous Asian chicken flocks [6–11]. Different from ALV-A, B, C, D, J and K, ALV-E belongs to endogenous ALV. MDV can be clustered into different pathotypes, including mild (m), virulent (v), very virulent (vv) and very virulent plus (vv+) strains. Marek’s disease (MD) caused by MDV is mainly characterized by lymphoproliferative disease in chickens with multiple neuritis or malignant tumors of the internal organs [12]. Except for inducing tumors, the infection of ALV or MDV also causes immunosuppression, which significantly affects the sustaining development of the poultry industry globally [13]. In China, although the ALV eradication program and the MD vaccination strategy greatly reduce the disease burdens caused by the infection of ALV and MDV, the frequent emergence of the novel ALV-K or the very virulent MDV (vvMDV) in the vaccinated chicken flock challenges the current control strategies for both diseases [2, 13, 14]. In this study, an outbreak of vvMDV infection in a vaccinated indigenous chicken flock co-infected with ALV-A, ALV-J and ALV-K was reported in China.

## Results

### Clinical symptoms and pathological changes

**Fig. 1:**
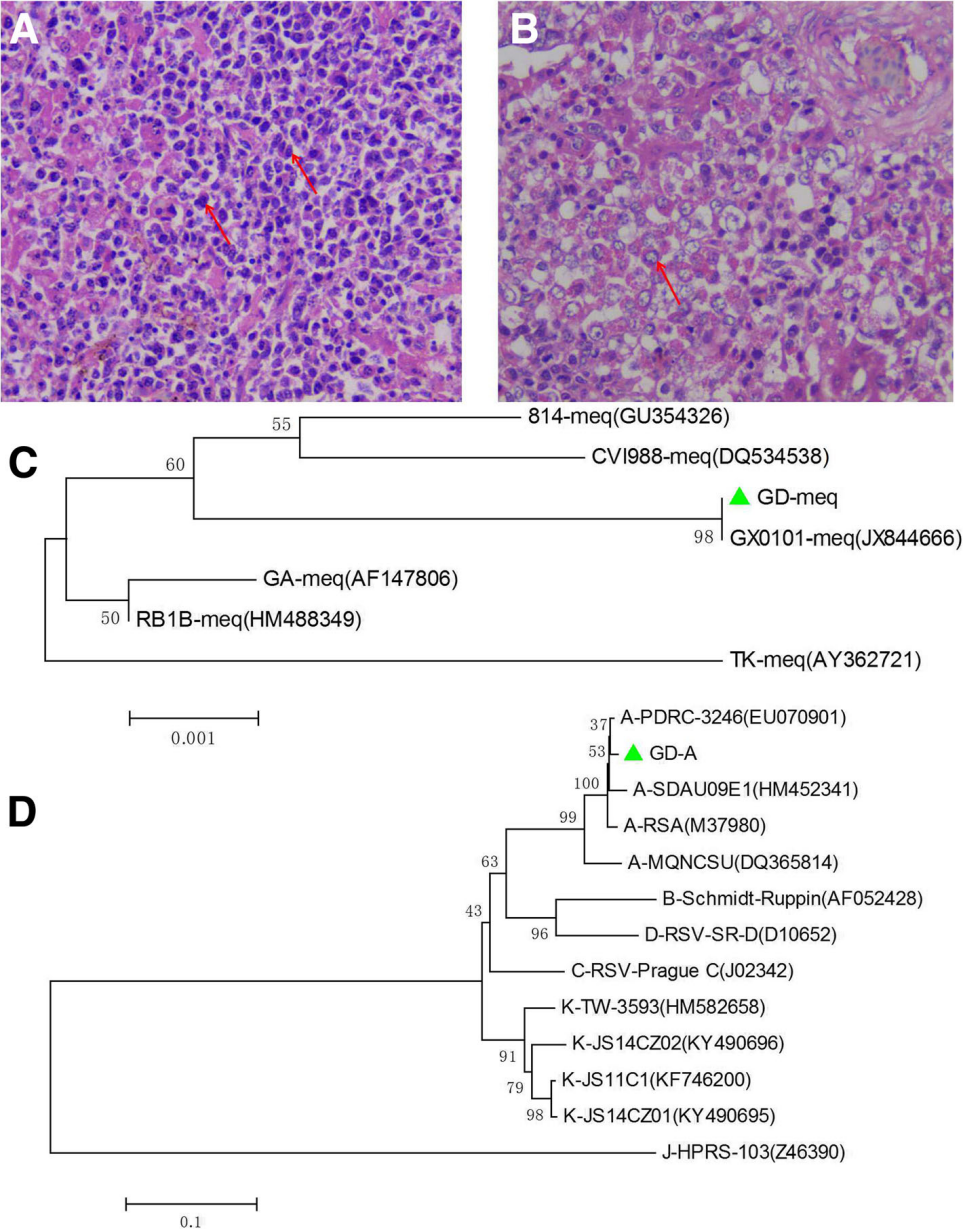
Pathological and molecular analysis for ALV-A and virulent MDV infection in the diseased chicken. **a**, **b** Pathological analysis for liver tissue of diseased chickens; **c**, **d** Phylogenic analysis for MDV-meq and ALV-A gp85 sequence, respectively

In 2017, a chicken farm in Guangdong Province suffered from neoplastic disease. The histopathological assay demonstrated that a large number of multinuclear tumor cells were observed in the liver tissue with pathological mitotic features (Fig. 1a), and many lymphocytic leukemia cells were found in the liver tissues with vacuolated nuclei (Fig. 1b). The histopathological assay indicated that these chickens might be infected with ALV and MDV.

### Co-infection of MDV with ALV-A, J and K

**Fig. 2:**
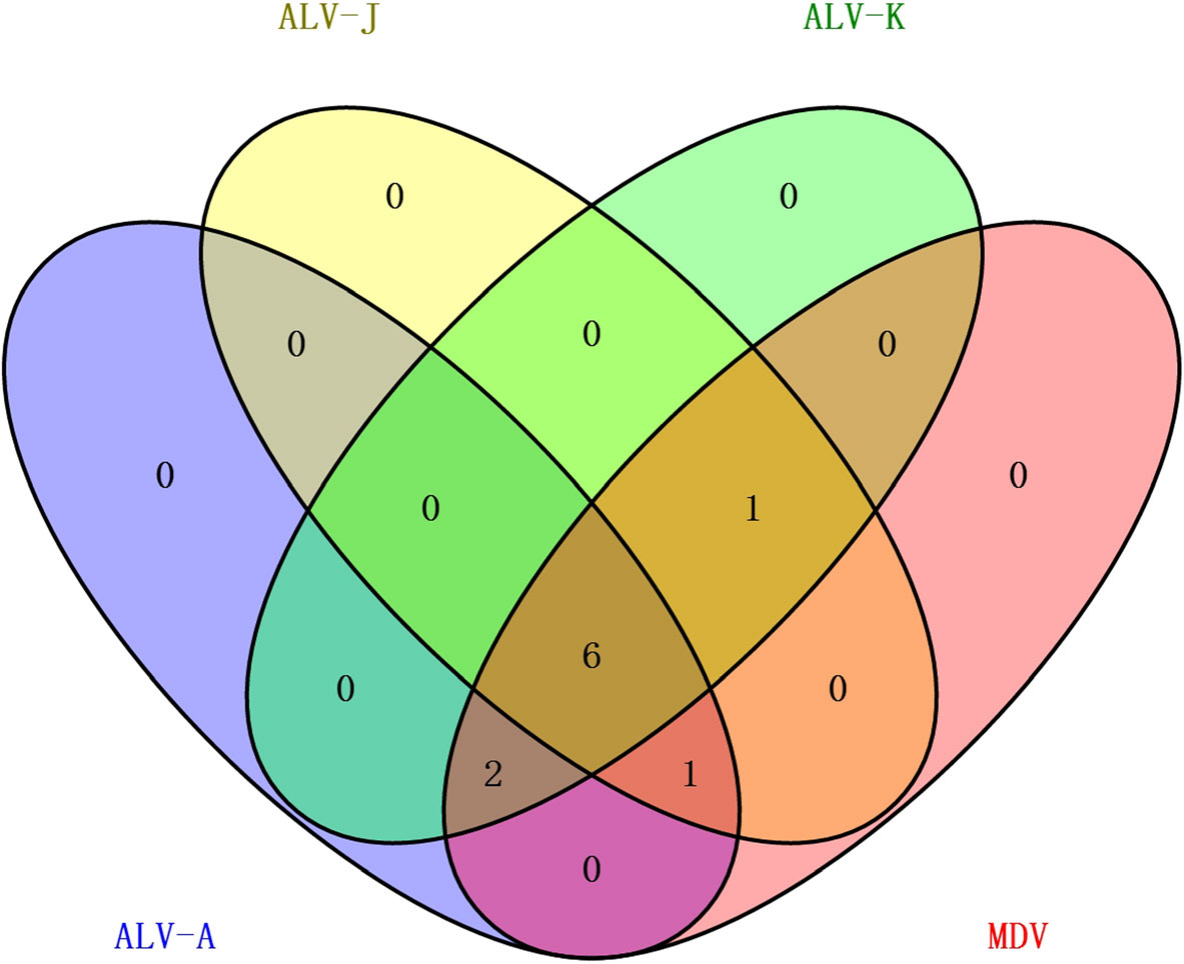
A Venn diagram showing the PCR detection results of Marek’s disease virus (MDV) and three subgroups of avian leukosis virus (ALV) in the diseased chickens. The red box shows chickens infected with MDV; the blue box shows chickens infected with ALV-A; the yellow box shows chickens infected with ALV-J; the green box shows chickens infected with ALV-K; the overlaps of different boxes show the co-infection status of the diseased chickens

To identify the potential causative agents, the tissue specimens (liver or spleen) from the diseased chickens were randomly collected, and the genomic DNA was extracted from these samples. Polymerase chain reaction (PCR) was used to detect oncogenic pathogens: ALV-A, ALV-B, ALV-J, ALV-K, Reticuloendotheliosis virus (REV) and MDV using the primers listed in Table 1. As described in Fig. 2, all the samples tested were positive for MDV in PCR but negative for REV and ALV-B. For the detection of ALV-A, ALV-J and ALV-K, the positive rate in 10 diseased chicken samples was 90, 80 and 90%, respectively (Fig. 2). Notably, all the PCR data were confirmed by sequencing the PCR products. These data clearly demonstrated that the diseased chicken flock was co-infected with MDV, ALV-A, ALV-J and ALV-K.

**Table 1:** Primers for PCR amplification of oncogenic pathogens

Pathogens	Primer sequence 5’→3’	Length (bp)	Reference
ALV-ABJK-F	ACCCGGAGAAGACACCCTT	–	This paper
ALV-A-R	AGGGGTGTCTAAGGAGAAACCG	563	This paper
ALV-B-R	CTGGGTCGGTCAGAAGGATGT	563	This paper
ALV-J-R	CATAGGGCCTTATAAGAAGGTCAT	563	This paper
ALV-K-R	TATAGCGGAGGAGGAGCCACCTCGT	559	This paper
REV	F: TGAGGGAAAATGTCATGCAACATCC R: ATCCCTACCCCACCCAGTAG	204	Davidson et al., 1995 [15]
MDV-meq	F: CGCGAATTCTACAGGTGTAAAGAGATG R: TAACTCGAGTGCTGAGAGTCACAATGC	1058	Zhuang et al., 2015 [16]
MDV-gB	F: CAGTCGACTATGCACTATTTTAG R: CAGGAATTCACAAGGAAAGCATCG	2800	Zhuang et al., 2015 [16]
MDV-pp38	F: AATGGATCCATGGAATTCGAAGCAGAAC R: ATTGTCGACAACATCGGGTACGGCTAC	903	Zhuang et al., 2015 [16]
ALV-K-A	F:ATCGATTGTAGTCAAATAGAGCCAGAGGC R: CATGGGAATTCCCCCTCCTATC	3558	This paper
ALV-K-B	F: ATCGATGATAGGAGGGGGAATTCCCATG R: GTCGACCTAGAGGGTACCCAAATAACC	2089	This paper
ALV-K-C	F: GGTTATTTGGGTACCCTCTCG R: GTCGACTGAAGCCTTCTGCTTCATTCAG	1881	This paper

### The identified MDVs belonged to vvMDV strains endemic in China

To further investigate whether the MDV detected in the diseased chicken flock is a vaccine strain or vvMDV strain, the *meq*, *gB* and *pp38* genes were amplified from these samples by PCR and analyzed. The sequence assay for the PCR products revealed that the *meq*, *gB* and *pp38* genes of the MDV (named GD) were 100% identical to the domestic vvMDV strain GX0101, which has been reportedly circulating on chicken farms in China for a long time (Fig. 1c) (gB and pp38 data not shown). The finding of vvMDV in this vaccinated chicken flock challenges the current MD vaccination strategy in China.

### ALV-A and ALV-K were efficiently isolated from the clinical samples

**Fig. 3:**
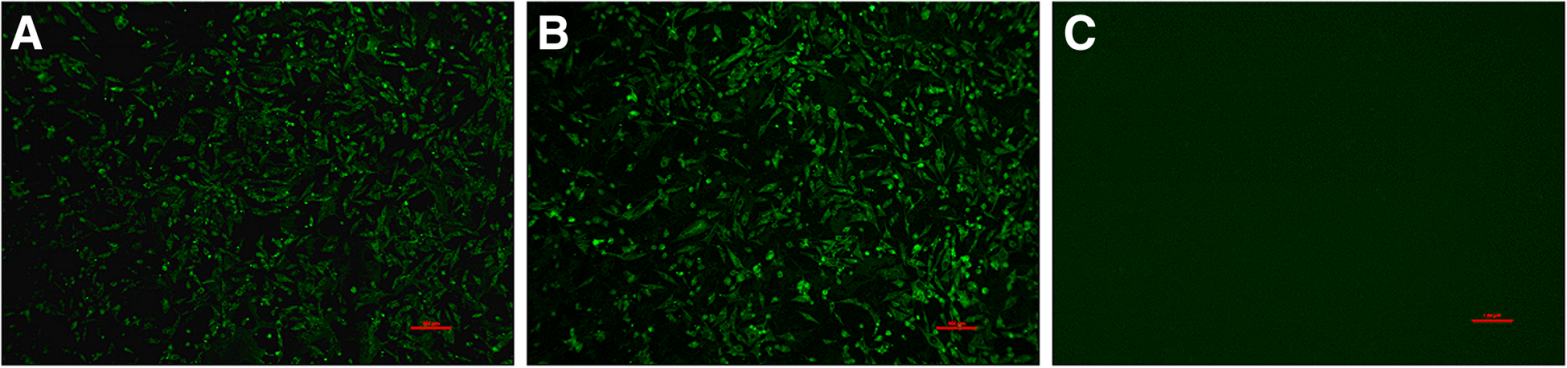
IFA for identifying ALV isolation. IFA for DF1 cells infected with the isolates using monoclonal antibody 5D3 specific to ALV-p27. DF1 cells infected with ALV-K GD-K and ALV-A GD-A (**a**, **b**); uninfected DF1 cells served as a negative control (**c**)

To further isolate ALVs in the diseased chickens, the homogenates of the PCR-positive samples for ALV-A, ALV-J and ALV-K were inoculated into DF1 cells. After several blind passages of the inoculated DF1 cells, the indirect immunofluorescent assay (IFA) and PCR were used to detect the isolation. Finally, one ALV-A isolate and one ALV-K isolate, named GD-A (Genbank accession No. MK951945) and GD-K (Genbank accession No. MK941182), respectively, were isolated from these samples and confirmed by IFA using monoclonal antibody (mAb) 5D3 against p27 (Fig. 3) and PCR with subgroup-specific primers (data not shown). The gp85 sequence of the GD-A isolate showed 94.4–99.1% identity with those reference ALV-A strains deposited in NCBI and lower than 86.9% similarity with other subgroups of ALV, which was confirmed by the phylogenetic analysis as described in Fig. 1d.

### The GD-K ALV-K isolate phylogenetically resembled the TW3593 isolate derived in Taiwan

**Fig. 4:**
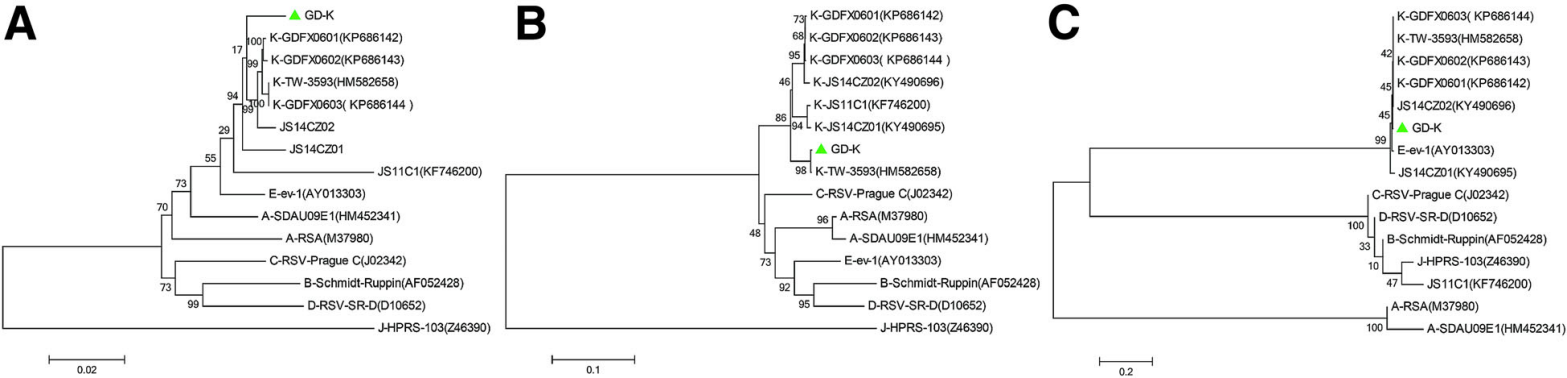
Genomic analysis for the isolated ALV-K strain. **a** Phylogenic analysis for GD-K complete genome; **b** Phylogenic analysis for gp85 sequence of GD-K **c** Phylogenic analysis for LTR sequence of GD-K

Since the ALV-K is a novel subtype of ALV recently identified in indigenous Asian chicken flocks [7, 8], to understand the molecular characteristics of the ALV-K isolate GD-K, the full genome of the GD-K was amplified by three overlapping rounds of PCR and sequenced. Sequence data showed that the complete genome was 7483 bp in length and had more than 97.6% identity with the ALV-K reference strains deposited in NCBI, except for JS11C (92.7%) (Fig. 4a). In addition, the gp85 sequence of GD-K showed 94.8–99.6% homology with the ALV-K reference strains and lower than 87.4% with other subgroups of ALVs (Fig. 4b). Notably, both the intact genome and gp85 sequence of the GD-K isolate demonstrated a close evolutionary relationship with the ALV-K isolate TW3593 from Taiwan (Fig. 4a and Fig. 4b). Further analysis of the long terminal repeat (LTR) sequence showed that the LTR of GD-K grouped together with TW-3593, CZ1401, CZ1402, GDFX0601, GDFX0602 and GDFX0603, as well as the ALV-E subgroup in the phylogenetic tree (Fig. 4c).

## Discussion

MDV and ALVs have caused severe economic losses to the poultry industry worldwide [17]. Notably, the frequent co-infection of MDV with ALVs or REV has undoubtedly become a great threat to the healthy development of the poultry industry [17–20]. In this study, vvMDV and ALV-K were first identified in a chicken flock in southern China. In the field, vvMDV or ALV-K single infection has been reported previously, which could cause immunosuppression in chickens and make them more susceptible to other pathogens. It should also be noted that ALV-K is frequently undetectable due to its poor replication ability [8, 10, 20–22]. The frequent transportation and communication of the indigenous chickens in southern China and the difficulties in detecting ALV-K were some other possible reasons for co-infection status [20].

In summary, a co-infection of vvMDV with multiple ALV subgroups was identified in a chicken flock with neoplastic disease in Guangdong Province. Moreover, subgroups ALV-A and ALV-K were efficiently isolated from two samples. To our knowledge, this is the first demonstration of co-infection of vvMDV with the novel ALV subgroup ALV-K. Notably, although the positive rate of ALV-J in these diseased chickens was 80% for PCR, ALV-J could not be efficiently isolated. This interesting finding indicated that co-infections with ALV-A or ALV-K, even vvMDV, might impact the replication of ALV-J. However, how these pathogens interact with each other remains to be further studied. In the case of ALV-A, resistant loci of *tva*_r1_, *t*va_r2_, *tva*_r3_, and *tv*a_r4_ have been found in some inbred lines of White Leghorn [23, 24]. In addition, single nucleotide polymorphism variants within *tva* receptor genes in some Chinese chicken breeds have been reported and animals with certain *tva* alleles are resistant to ALV-A infection [25, 26]. Notably, a recent research has showed that the novel ALV-K shares its *tva* cell receptor with ALV-A [27]. Thus, the *tva* receptors in the chicken flock are possibly associated with ALV-A and ALV-K infection status in this study as well. In short, future study should also focus on the polymorphisms of *tva* receptors in different indigenous chicken breeds in China, test the susceptibility of these indigenous chickens to ALV-A and ALV-K infections, and breed chickens resistant to ALV-A and ALV-K.

## Conclusions

The co-infection of vvMDV with different subgroups of ALV identified in a chicken flock poses a risk for the emergence of novel ALVs and burdens the control strategy for MD and highlights the significance of epidemiological monitoring for similar co-infection in indigenous chicken flocks in China.

## Methods

### Clinical samples

Layer chickens that were 150 days old and vaccinated with MDV and suffering from neoplastic disease with about 10% neoplastic incidence and 5% mortality were obtained from a large chicken farm in Guangdong Province, China. The owner of the farm gave permission to include ten diseased chickens in this study. Tumor nodules were found on the surface of the organs and skin from these diseased chickens. All experiments complied with institutional animal care guidelines and were approved by the University of Yangzhou Animal Care Committee.

### Histopathological assay

A histopathological assay was conducted as previously described to examine the tissues [16]. Briefly, the livers from the diseased chickens were fixed using 10% formalin buffer, dehydrated in alcohol, and embedded in paraffin. Hematoxylin and eosin staining was then performed, and microscopic changes were observed by light microscopy.

### PCR detection for oncogenic pathogens

Genomic DNA from the tissues of diseased chickens was first extracted and detected with specific primers (Table 1) for the oncogenic pathogens MDV, ALV and REV by PCR [15, 16].

### Virus isolation

The homogenates of the PCR-positive tissue samples were filtered through a 0.22 μm filter and inoculated into DF1 cells for 2 h. Then, fresh Dulbecco’s modified Eagle’s medium (DMEM) with 1% fetal bovine serum (FBS) was used for replacement. The supernatant of the cell culture was passed for three serial passages (5–7 days for each passage), and then the infected cells were screened for ALV by PCR and IFA.

### Indirect immunofluorescence assay

The infected DF1 cells were fixed with chilled acetone:ethanol solution (3:2) for 5 min and washed once with PBS. Then, they were incubated with the ALV-p27-specific mAb 5D3 for 45 min at 37 °C [28]. After three washes with PBS, the cells were incubated with fluorescein isothiocyanate (FITC)-conjugated goat anti-mouse antibody for another 45 min. After three washes with PBS, the cells were observed under a fluorescence microscope.

### Sequence analysis

For PCR, the gB, pp38 and meq genes were amplified as described [16]. For amplification of ALV and REV genes, 50 μl of reaction volume was used, which consisted of 10 μl of 5 × SF PCR buffer, 1 μl dNTP mixture, 2 μl of each primer, 1 μl of Phanta Super-Fidelity DNA Polymerase (Vazyme, Nanjing, China), 32 μl of ddH_2_O, and 2 μl of the DNA template. The PCR programs for the pp38 and meq genes were 94 °C for 3 min, 35 cycles of 94 °C for 30 s, 55 °C for 30 s, and 72 °C for 1 min 30 s, and then 72 °C for 10 min. The PCR products were separated by 1% agarose gel electrophoresis and then sequenced by Genscript (Nanjing, China). All sequences were aligned with Lasergene 7 and phylogenetically analyzed with MEGA 6.

## Data Availability

The datasets used and/or analysed during the current study are available from the corresponding author on reasonable request.

## Abbreviation

ALV: Avian leukosis virus
ALV-A: Avian leukosis virus subgroup A;
DMEM: Dulbecco’s modified Eagle’s medium
FBS: Fetal bovine serum
FITC: Fluorescein isothiocyanate
IFA: Indirect immunofluorescent assay
LTR: Long terminal repeat
MD: Marek’s disease
MDV: Marek’s disease virus
PCR: Polymerase chain reaction
REV: Reticuloendotheliosis virus
v: Virulent
vv: Very virulent
vv+: Very virulent plus
vvMDV: Very virulent MDV
